# Integrating neuroimaging and plasma biomarkers to predict preclinical Alzheimer’s disease progression

**DOI:** 10.3389/fneur.2026.1801239

**Published:** 2026-05-05

**Authors:** Junjia Qi, Leishen Li, Hongyan Duan, Yajing Sun, Jiewen Zhang

**Affiliations:** 1 Department of Geriatrics, People’s Hospital of Zhengzhou University/Henan Provincial People’s Hospital, Zhengzhou, China; 2 Department of Neurology, People’s Hospital of Zhengzhou University/Henan Provincial People’s Hospital, Zhengzhou, China

**Keywords:** Alzheimer’s disease, disease progression, machine learning, multimodal fusion, preclinical stage, prediction model

## Abstract

**Objective:**

To develop and validate a multimodal model integrating neuroimaging and plasma biomarkers for predicting the risk of cognitive progression in preclinical Alzheimer’s disease (AD).

**Methods:**

This retrospective study enrolled 320 patients with Aβ-positive preclinical AD or AD-related mild cognitive impairment. Participants were randomly allocated into training and validation sets at a 7:3 ratio. In the training set, univariable analysis, least absolute shrinkage and selection operator (LASSO) regression, and multivariable Logistic regression were employed to identify core predictive variables. Subsequently, four machine learning models were constructed based on these variables. Model performance was evaluated using the area under the receiver operating characteristic Curve (AUC), calibration curves, and decision curve analysis. Interpretability was assessed using SHapley Additive exPlanations (SHAP) values.

**Results:**

The baseline characteristics were balanced between the training and validation sets. LASSO regression identified five core variables: Mini-Mental State Examination total score, Rey Auditory Verbal Learning Test delayed recall, normalized hippocampal volume, plasma phosphorylated tau181, and apolipoprotein E ε4 allele status. Multivariable analysis confirmed these as independent predictors (*p* < 0.01). The logistic regression model demonstrated robust predictive performance, achieving the highest area under the curve (AUC) in the independent validation set (0.818, 95% confidence interval: 0.703–0.933). Calibration and decision curve analyses conducted on the validation set indicated that the model was accurate and possessed clinical utility. SHAP analysis applied to the optimal model showed that normalized hippocampal volume was the most influential contributor to the predictions.

**Conclusion:**

The developed multimodal model exhibits robust predictive performance and clinical utility. It may serve as a quantitative tool for individualized risk management in preclinical AD.

## Introduction

Alzheimer’s disease (AD) is the leading cause of dementia, with its pathological processes initiating more than a decade before the onset of clinical symptoms (1, 2). The preclinical AD (amyloid-beta (Aβ)-positive, cognitively unimpaired) and AD-related mild cognitive impairment (MCI) are consecutive early stages of the AD pathological process, both characterized by Aβ positivity as the core pathological feature, and also serve as critical time windows for intervening in AD disease progression. However, the risk of progression to dementia during this stage is highly heterogeneous (3). Consequently, the early and precise prediction of which individuals will progress rapidly is crucial for implementing targeted interventions and optimizing clinical trial enrollment. Although individual biomarkers, such as Aβ-positron emission tomography (Aβ-PET) or plasma phosphorylated-tau (p-tau), are effective for diagnosing AD pathology, their ability to predict the rate of progression at the individual level is limited (4). AD progression involves complex interactions across multiple pathophysiological dimensions, including amyloid deposition, tau tangles, neurodegeneration, and cognitive reserve. Therefore, integrating multimodal biomarkers that reflect these different pathological dimensions, such as neuroimaging and plasma biomarkers, holds promise for constructing more accurate predictive models (5). Machine learning methods, capable of handling high-dimensional data and capturing complex variable relationships, are ideal tools for building such integrated models (6). Nevertheless, a rigorously validated predictive model for preclinical AD progression, systematically integrating neuroimaging and emerging plasma biomarkers within a Chinese population, is currently lacking (7). To address this gap, this retrospective cohort study collected neuropsychological, neuroimaging (Aβ-PET, structural magnetic resonance imaging [MRI]), plasma biomarker, and genetic data. Utilizing feature selection and machine learning algorithms, this study aims to develop and validate a high-performance, interpretable, multimodal fusion prediction model to provide decision support for the early and precise prevention and management of AD.

## Materials and methods

### Study population

This retrospective cohort study consecutively enrolled 320 participants from an AD research center who met the criteria for preclinical AD or MCI and were Aβ-positive between January 1, 2019 and December 31, 2022. The study protocol was approved by the institutional ethics review board, and written informed consent was obtained from all participants or their legal guardians.

The sample size was calculated based on preliminary experimental data and literature reports, with an expected incidence of the primary outcome (cognitive progression) of 20–25%. Estimation was performed using PASS 2021 software supplemented by the “pwr” package in R (version 4.2.3). With a significance level of *α* = 0.05 (two-tailed), a statistical power (1 − *β*) of 80%, and an anticipated data missing rate of 5%, the minimum required sample size was determined. Adhering to the empirical rule of at least 10 outcome events per estimated parameter in predictive model development, and considering approximately 5–6 core variables, the calculation yielded a minimum required sample size of 250–300 participants. The final enrollment of 320 participants provided a validated statistical power exceeding 85%, meeting the requirements for multimodal variable analysis and machine learning modeling. Cognitive status at the last available follow-up visit was used to determine the primary outcome (progression versus stable).

Inclusion criteria: (1) Age ≥60 years; (2) Meeting internationally recognized diagnostic criteria for preclinical AD (Aβ-positive confirmed by Aβ-PET and cognitively unimpaired) or MCI with an etiological suggestion of AD (Aβ-positive); (3) Complete baseline cognitive assessment data; (4) Availability of baseline Aβ-PET, structural MRI, and plasma biomarker data; (5) Planned for long-term follow-up, with complete baseline and follow-up neuropsychological assessment data. (5) Had at least one follow-up cognitive assessment, allowing for determination of progression status. A total of 400 potentially eligible participants were initially recruited for this study between January 1, 2019, and December 31, 2022. After individual review, participants meeting any of the following criteria were excluded: (1) Presence of other neurological diseases that could cause dementia or cognitive decline; (2) History of severe mental illness or substance abuse; (3) Presence of severe systemic diseases that could affect cognitive assessment or life expectancy; (4) Negative baseline Aβ-PET scan result; (5) Contraindications for MRI or poor image quality; (6) Lack of follow-up data on disease progression status. Finally, 320 participants were included in the subsequent analysis.

### Data collection

Participant information was collected from the research center’s electronic medical record system, neuroimaging database, and biobank, covering the following domains:

#### Demographics and basic clinical data

Age, sex, years of education, history of hypertension, history of diabetes, family history of AD.

#### Clinical staging and neuropsychological assessment

Clinical stage (preclinical AD/MCI). Comprehensive cognitive assessment was performed using the Mini-Mental State Examination (MMSE), the Alzheimer’s Disease Assessment Scale-Cognitive Subscale 13-item version (ADAS-Cog13), the Rey Auditory Verbal Learning Test (RAVLT) delayed recall, and Trail Making Test part B (TMT-b) time.

#### Neuroimaging biomarkers

Cerebral Aβ deposition was assessed using positron emission tomography and expressed as the standardized uptake value ratio. High-resolution T1-weighted structural images were acquired using 3.0 T magnetic resonance imaging. Post-processing with software such as FreeSurfer was used to extract normalized hippocampal volume, medial temporal lobe cortical thickness, mean global cortical thickness, and white matter hyperintensity volume.

#### Plasma biomarkers

Fasting peripheral blood was collected. After plasma separation, concentrations of p-tau181, the Aβ 42/40 ratio, neurofilament light chain, and glial fibrillary acidic protein were quantified using Single Molecule Array technology.

#### Genetic indicators

Peripheral blood samples were collected for genomic DNA extraction. Apolipoprotein E (APOE) genotype was determined using the probe amplification or gene chip method to identify APOE ε4 allele carrier status. An AD polygenic risk score was calculated based on large-scale genome-wide association study results.

### Grouping criteria

The diagnostic definitions of preclinical AD and AD-related MCI in this study were consistent throughout the entire enrollment and analysis process; preclinical AD was defined as Aβ-PET positive with unimpaired cognitive function, and AD-related MCI was defined as Aβ-PET positive with mild cognitive impairment. Based on this, the group determination of disease progression and stable groups was carried out, with the primary study endpoint being cognitive status progression during long-term follow-up.

#### Disease progression group

Participants with baseline preclinical AD who progressed to MCI or dementia during follow-up, or participants with baseline MCI who progressed to dementia. All progression was determined by clinicians according to international diagnostic criteria.

#### Disease stable group

Participants whose cognitive status remained at the baseline level throughout the follow-up period (preclinical AD remained cognitively unimpaired; MCI did not progress to dementia).

#### Adjudication process and blinding

After the follow-up period, research assistants, blinded to all baseline multimodal biomarker data, conducted neuropsychological assessments. All clinical and assessment data were entered into a database. Two independent neurologists or neuropsychologists then adjudicated the progression status based on pre-defined diagnostic criteria. In case of disagreement, a third senior expert arbitrated to determine the final grouping.

### Statistical analysis

All data analyses were completed using SPSS 26.0, R 4.2.3 and Python 3.8.5; core variables were first screened by traditional statistical methods and their independent predictive value was verified, then machine learning models were constructed for comparative analysis. Normally distributed continuous data are presented as mean ± standard deviation and compared using independent samples *t*-tests. Non-normally distributed data are presented as median (interquartile range) and compared using the Mann–Whitney *U* test. Categorical data are presented as number (percentage) and compared using the *χ*^2^ test or Fisher’s exact test.

All participants were randomly split into a training set and a validation set at a 7:3 ratio. In the training set, univariable analysis was first conducted to screen variables associated with disease progression. Variables with statistical significance (*p* < 0.05) in the univariable analysis were considered candidate predictors. To handle high-dimensional data and select the most predictive core variables, all candidate predictors were entered into a least absolute shrinkage and selection operator (LASSO) regression model for feature selection. The optimal penalty coefficient (*λ*.min) was determined via 10-fold cross-validation. This *λ* value corresponds to the set of variables with non-zero coefficients when the model’s mean squared error is minimized during cross-validation, constituting the selected core predictor set. Subsequently, the core variables selected by LASSO were included in a multivariable logistic regression analysis to calculate their odds ratios (ORs) and 95% confidence intervals (CIs), further confirming their value as independent influencing factors. Collinearity diagnosis was performed using the variance inflation factor (VIF) and correlation matrix.

Based on the core variables selected by LASSO, four machine learning prediction models were constructed: logistic regression, support vector machine, random forest, and gradient boosting machine. Optimal hyperparameter tuning for all machine learning models was completed in the training set by grid search combined with 5-fold cross-validation, with the specific tuning details as follows: logistic regression tuned the regularization strength *C* (0.01–10) with the optimal *C* = 1; support vector machine (radial basis kernel) tuned *C* (0.01–10) and gamma (0.001–1) with the optimal *C* = 2 and gamma = 0.1; random forest tuned the number of decision trees (100–500) and maximum depth (5–20) with the optimal number of 200 and depth of 10; gradient boosting machine tuned the number of decision trees (100–500), learning rate (0.01–0.1) and maximum depth (3–10) with the optimal number of 300, learning rate of 0.05 and depth of 5. The area under the operating characteristic (ROC) curve (AUC) was used to evaluate model discrimination. Calibration curves were plotted to assess the agreement between predicted probabilities and observed frequencies. Decision curve analysis was performed to evaluate the clinical net benefit across different risk thresholds. A visual nomogram was constructed and internally validated using the bootstrap method (1,000 repetitions). Finally, the SHAP (SHapley Additive exPlanations) value framework was applied to the best-performing model for interpretability analysis, elucidating the contribution strength of each feature to the prediction outcome from a global perspective. All statistical tests were two-sided, with *p* < 0.05 considered statistically significant.

## Results

### Comparison of baseline characteristics between training and validation sets

No statistically significant differences (*p* > 0.05) were observed between the training and validation sets regarding demographic characteristics (age, sex, years of education), clinical history (hypertension, diabetes, family history of AD), clinical staging, neuropsychological scores (MMSE, ADAS-Cog13, RAVLT, TMT-b), neuroimaging biomarkers (Aβ-PET standardized uptake value ratio [SUVR], hippocampal volume, cortical thickness, etc.), plasma biomarkers (p-tau181, Aβ42/40, neurofilament light chain [NfL], glial fibrillary acidic protein [GFAP]), or genetic characteristics (APOE ε4 status, polygenic risk score). The dataset split was balanced, indicating good comparability (Table 1).

**Table 1:** Comparison of baseline characteristics between training and validation sets.

Variables	Training set (*n* = 224)	Validation set (*n* = 96)	*t*/*χ*^2^	*p*
Age (years)	71.30 ± 5.90	72.00 ± 5.60	0.987	0.324
Sex			0.381	0.537
Male	92 (41.07)	43 (44.79)		
Female	132 (58.93)	53 (55.21)		
Years of education	12.90 ± 3.30	12.50 ± 3.00	1.020	0.308
History of hypertension (%)			0.173	0.678
Yes	118 (52.68)	53 (55.21)		
No	106 (47.32)	43 (44.79)		
History of diabetes (%)			0.325	0.569
Yes	45 (20.09)	22 (22.92)		
No	179 (79.91)	74 (77.08)		
Family history of AD (%)			0.368	0.544
Yes	67 (29.91)	32 (33.33)		
No	157 (70.09)	64 (66.67)		
Clinical stage (%)			0.060	0.807
Preclinical AD (Aβ+, cognitively normal)	106 (47.32)	44 (45.83)		
MCI due to AD	118 (52.68)	52 (54.17)		
MMSE total score	26.90 ± 2.00	26.60 ± 2.30	1.174	0.241
ADAS-Cog13 total score	12.30 ± 4.20	13.00 ± 4.50	1.337	0.182
RAVLT delayed recall	5.20 ± 2.10	5.00 ± 2.30	0.758	0.449
TMT-b time, s	125.60 ± 45.30	130.40 ± 48.10	0.853	0.395
Aβ-PET SUVR	1.22 ± 0.18	1.25 ± 0.17	1.389	0.166
Normalized hippocampal volume, mL	3.14 ± 0.44	3.08 ± 0.47	1.095	0.274
Medial temporal lobe cortical thickness, mm	2.66 ± 0.27	2.63 ± 0.30	0.881	0.379
Mean global cortical thickness, mm	2.50 ± 0.15	2.48 ± 0.16	1.071	0.285
White matter hyperintensity volume, mL	1.05 ± 0.52	1.09 ± 0.54	0.623	0.534
Plasma p-tau181, pg/mL	2.77 ± 1.48	2.85 ± 1.53	0.439	0.661
Plasma Aβ42/40 ratio	0.05 ± 0.01	0.05 ± 0.01	0.001	1.000
Plasma NfL, pg/mL	18.80 ± 7.90	19.40 ± 8.20	0.616	0.539
Plasma GFAP, pg/mL	185.50 ± 86.30	192.10 ± 90.50	0.618	0.537
APOE ε4 allele status			0.239	0.625
Carrier (%)	121 (54.02)	49 (51.04)		
Non-carrier (%)	103 (45.98)	47 (48.96)		
Polygenic risk score	0.12 ± 0.94	0.21 ± 0.97	0.777	0.438

AD, Alzheimer’s disease; Aβ, amyloid-beta; MCI, mild cognitive impairment; MMSE, Mini-Mental State Examination; ADAS-Cog13, Alzheimer’s Disease Assessment Scale-Cognitive Subscale 13-item version; RAVLT, Rey Auditory Verbal Learning Test; TMT-b, Trail Making Test part B; Aβ-PET SUVR, amyloid-beta positron emission tomography standardized uptake value ratio; p-tau, phosphorylated-tau; NfL, neurofilament light chain; GFAP, glial fibrillary acidic protein; APOE, apolipoprotein E.

### Comparison of baseline characteristics between the disease progression and stable groups in the training set

Within the training cohort of 224 participants, individuals were stratified into a Disease Progression group (*n* = 56) and a Disease Stable group (*n* = 168) based on cognitive status changes during follow-up. Univariate analysis showed that the Disease Progression group had significantly lower baseline MMSE total scores and AVLT delayed recall scores. Conversely, this group demonstrated significantly higher Aβ-PET SUVR, higher plasma p-tau181 levels, smaller standardized hippocampal volumes, and a higher proportion of APOE ε4 allele carriers (*p* < 0.05). No statistically significant differences were observed between the two groups regarding age, sex, years of education, medical history, ADAS-Cog13 total score, or other metrics (*p* > 0.05) (Supplementary Table 1).

### Variable selection via LASSO regression

**Figure 1:**
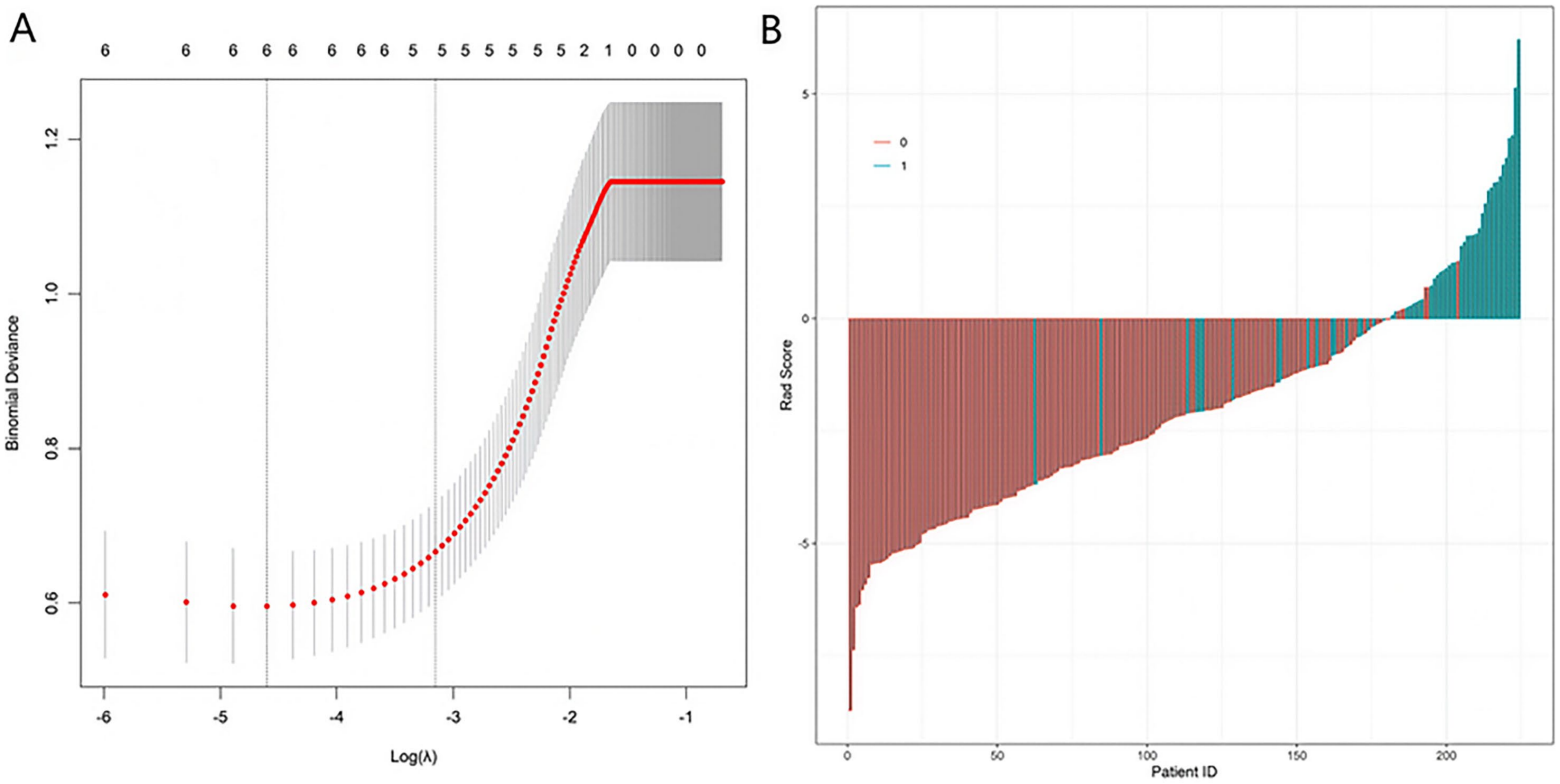
LASSO regression path plot **(A)** and coefficient profile plot **(B)**.

To identify core predictive variables and mitigate overfitting, the six significant variables (MMSE total score, RAVLT delayed recall, Aβ-PET SUVR, standardized hippocampal volume, plasma p-tau181, and APOE ε4 status) were incorporated into a LASSO regression analysis. The optimal penalty coefficient (*λ*) was determined using 10-fold cross-validation. The LASSO path plot illustrated that variable coefficients progressively shrank toward zero as the absolute value of log(*λ*) increased. The curve stabilized when log(*λ*) decreased to a specific value, indicating the optimal predictive performance of the variable combination at that point (Figure 1A). Ultimately, the LASSO regression retained five core predictors: MMSE total score, RAVLT delayed recall, standardized hippocampal volume, plasma p-tau181, and APOE ε4 allele status, suggesting that this 5-variable combination possesses robust predictive potential (Figure 1B). Correlation matrix analysis showed that the correlation coefficient between Aβ-PET SUVR and normalized hippocampal volume was −0.48, and that with plasma p-tau181 was 0.45. Aβ-PET SUVR was excluded from the final model.

### Multivariate logistic regression analysis of neuroimaging and plasma biomarkers for AD progression

The five variables selected by LASSO regression were entered into a multivariate logistic regression model. The results demonstrated that all five variables were independent influencing factors for disease progression (all *p* < 0.05). Specifically, for each 1-point decrease in the MMSE total score, the risk of disease progression increased by a factor of 1.67 (OR = 0.600, 95% CI: 0.470–0.764). Each 1-unit reduction in RAVLT delayed recall increased the risk by a factor of 1.50 (OR = 0.667, 95% CI: 0.516–0.862). Furthermore, each 1 mL decrease in standardized hippocampal volume elevated the risk by a factor of 1.69 (OR = 0.593, 95% CI: 0.434–0.810). Each 1 pg./mL increase in plasma p-tau181 concentration was associated with a 1.39-fold increased risk (OR = 1.385, 95% CI: 1.190–2.633). Finally, individuals carrying the APOE ε4 allele had a 3.23-fold higher risk of disease progression compared to non-carriers (OR = 3.234, 95% CI: 1.292–8.099) (Table 2). The results multicollinearity analysis showed that the VIF of the five core variables were all <3 with no strong correlation (*r* < 0.7), confirming no significant multicollinearity.

**Table 2:** Multivariate logistic regression analysis of the five selected neuroimaging and plasma biomarkers for AD progression.

Variables	*β*	SE	Wald	OR (95% CI)	*p*
MMSE total score	−0.512	0.124	17.047	0.600 (0.470–0.764)	0.001
RAVLT delayed recall	−0.405	0.131	9.566	0.667 (0.516–0.862)	0.002
Standardized hippocampal volume	−0.522	0.158	10.901	0.593 (0.434–0.810)	0.001
Plasma p-tau181	0.647	0.164	15.598	1.385 (1.190–2.633)	0.001
APOE ε4 allele status	1.174	0.468	6.283	3.234 (1.292–8.099)	0.012
Constant	16.358	4.212	15.082	—	0.001

AD, Alzheimer’s disease; MMSE, Mini-Mental State Examination; RAVLT, Rey Auditory Verbal Learning Test; p-tau, phosphorylated-tau; APOE, apolipoprotein E.

### Machine learning model performance evaluation

**Figure 2:**
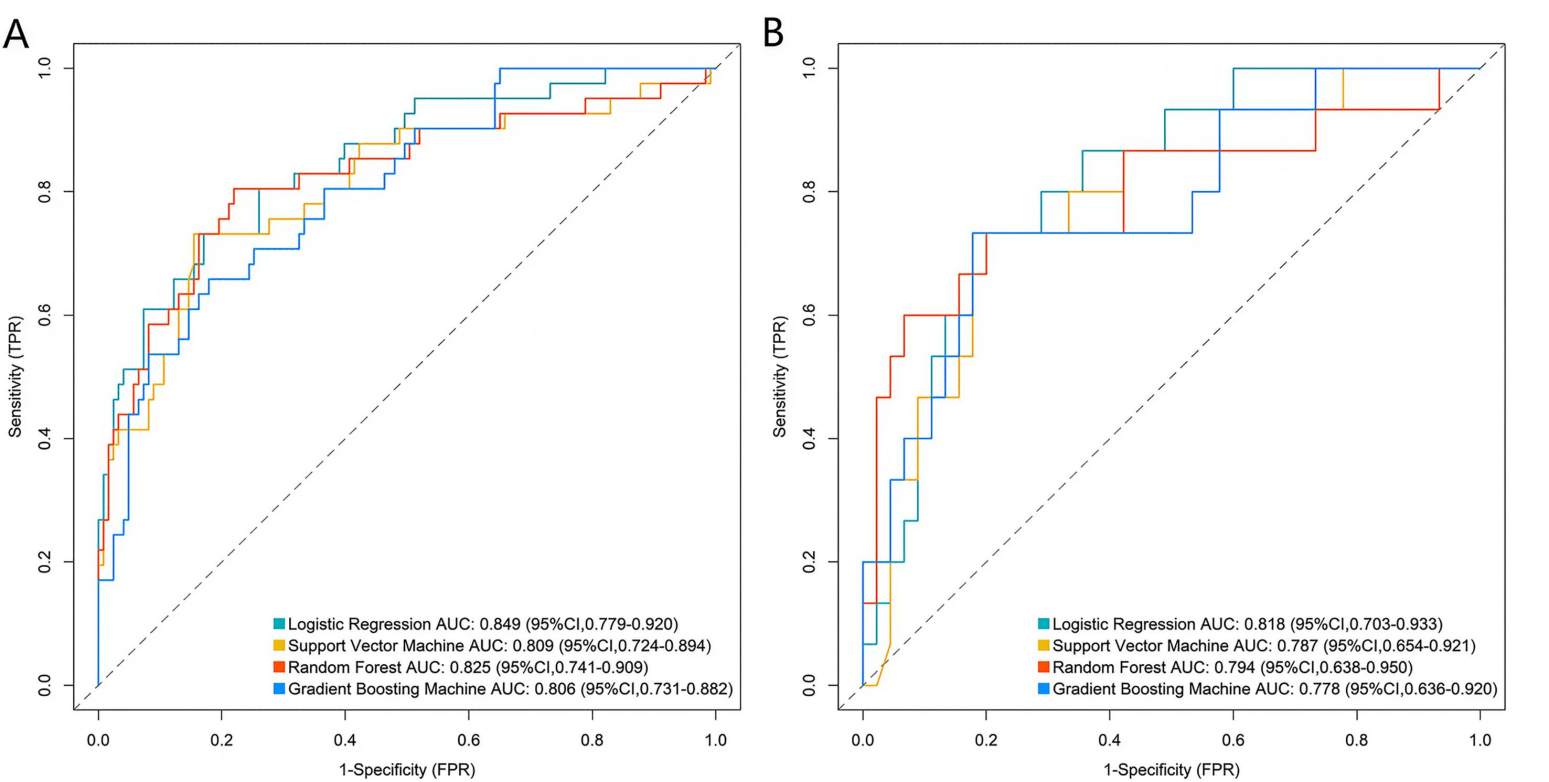
Receiver operating characteristic curve analysis of the prediction model in the training **(A)** and validation **(B)** sets.

**Figure 3:**
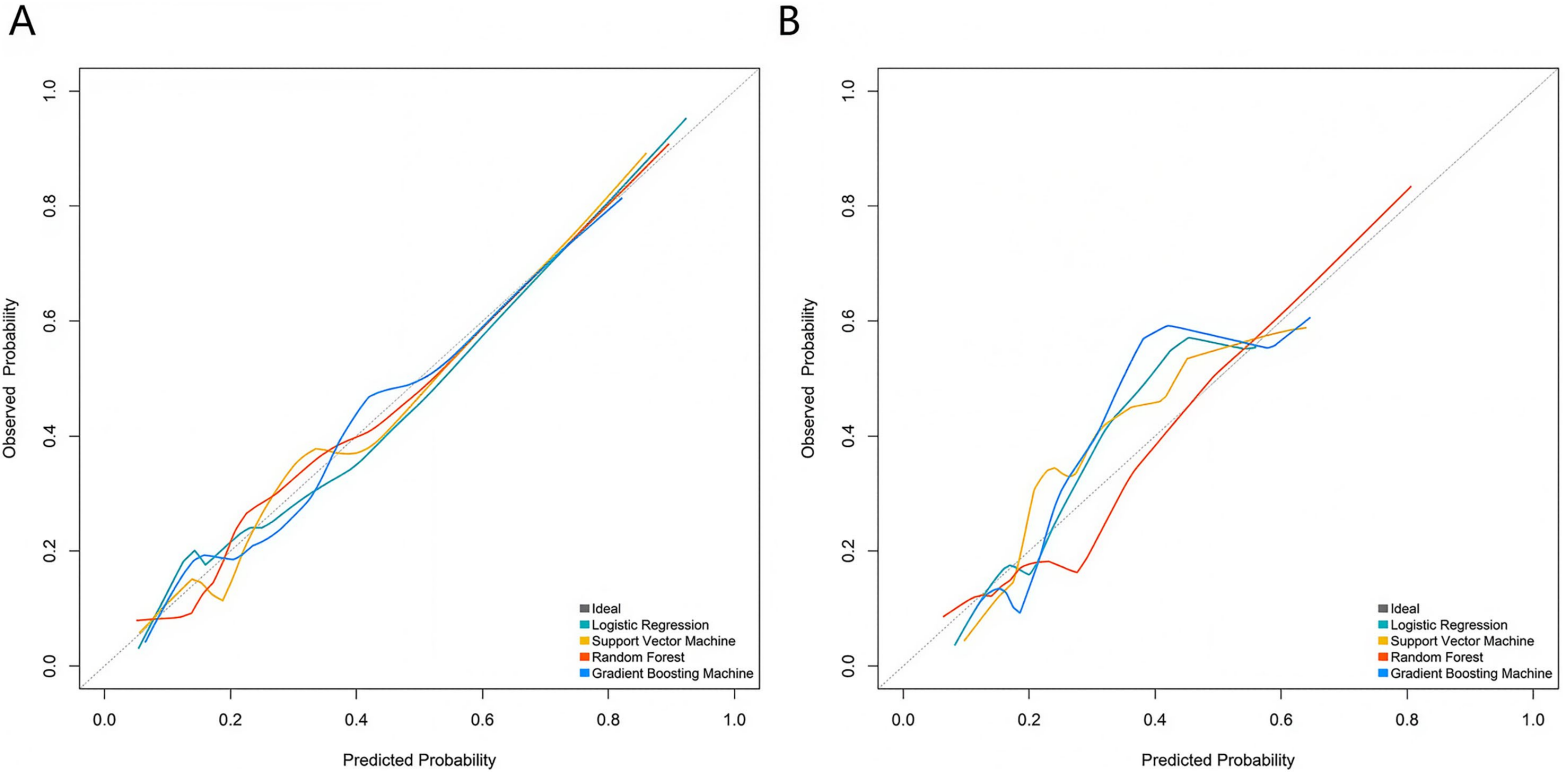
Calibration curves of the prediction model in the training **(A)** and validation **(B)** sets.

**Figure 4:**
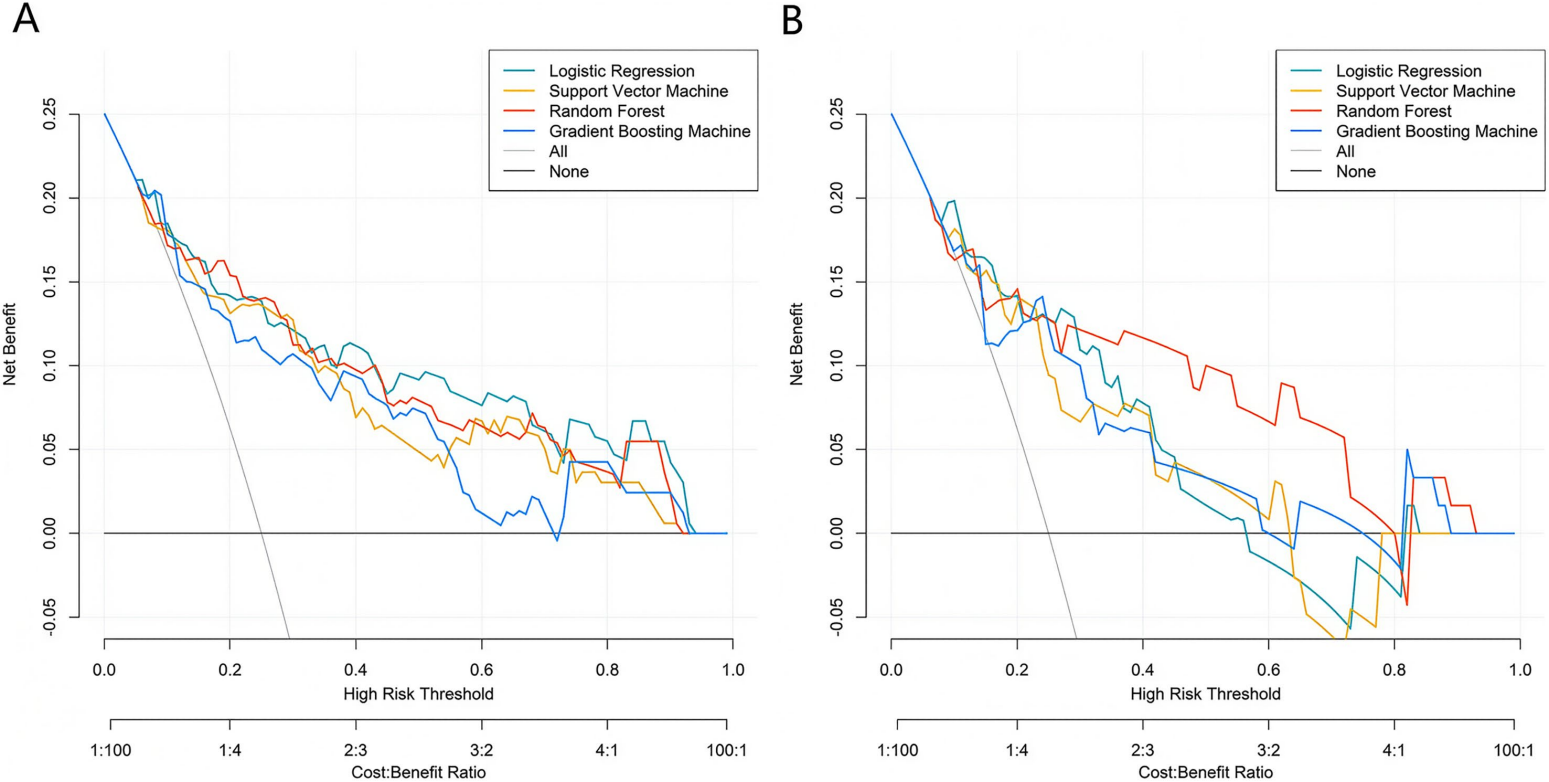
Clinical decision curve analysis of the prediction model in the training **(A)** and validation **(B)** sets.

Based on the five core variables, four machine learning models were constructed: logistic regression, support vector machine, random forest, and gradient boosting machine. ROC curve results indicated that, in the training set, the Logistic Regression model achieved the highest AUC value (0.849, 95% CI: 0.779–0.920), followed by random forest (0.825, 95% CI: 0.741–0.909), support vector machine (0.809, 95% CI: 0.724–0.894), and gradient boosting machine (0.806, 95% CI: 0.731–0.882). In the validation set, the logistic regression model again yielded the highest AUC (0.818, 95% CI: 0.703–0.933), followed by random forest (0.794, 95% CI: 0.638–0.950), support vector machine (0.787, 95% CI: 0.654–0.921), and gradient boosting machine (0.778, 95% CI: 0.636–0.920) (Figures 2A,B). The calibration curves demonstrated good overall agreement between predicted probabilities and observed probabilities across all models, indicating that the predicted risks were well-calibrated against actual outcomes (Figures 3A,B). Decision curve analysis (Figures 4A,B) showed that across a wide range of clinically relevant risk thresholds, the net benefit of all four models significantly exceeded the strategies of “treat all” or “treat none,” suggesting their potential clinical utility for individualized risk assessment. These findings collectively support the robustness and generalizability of the multimodal prediction framework, laying a foundation for future prospective validation and potential integration into clinical decision-support systems.

### Sensitivity analysis

To further verify the rationality of excluding Aβ-PET SUVR from the final logistic regression model, a sensitivity analysis was performed by comparing the performance of the 6-variable model (incorporating Aβ-PET SUVR) and the original 5-variable model in both the training and validation sets. The results showed that in the training set, the AUC of the 6-variable model was 0.842 (95% CI: 0.770–0.914), which was slightly lower than 0.849 (95% CI: 0.779–0.920) of the 5-variable model; in the validation set, the AUC of the 6-variable model was 0.812 (95% CI: 0.695–0.929), which was also lower than 0.818 (95% CI: 0.703–0.933) of the 5-variable model.

### Interpretability assessment of model predictions

**Figure 5:**
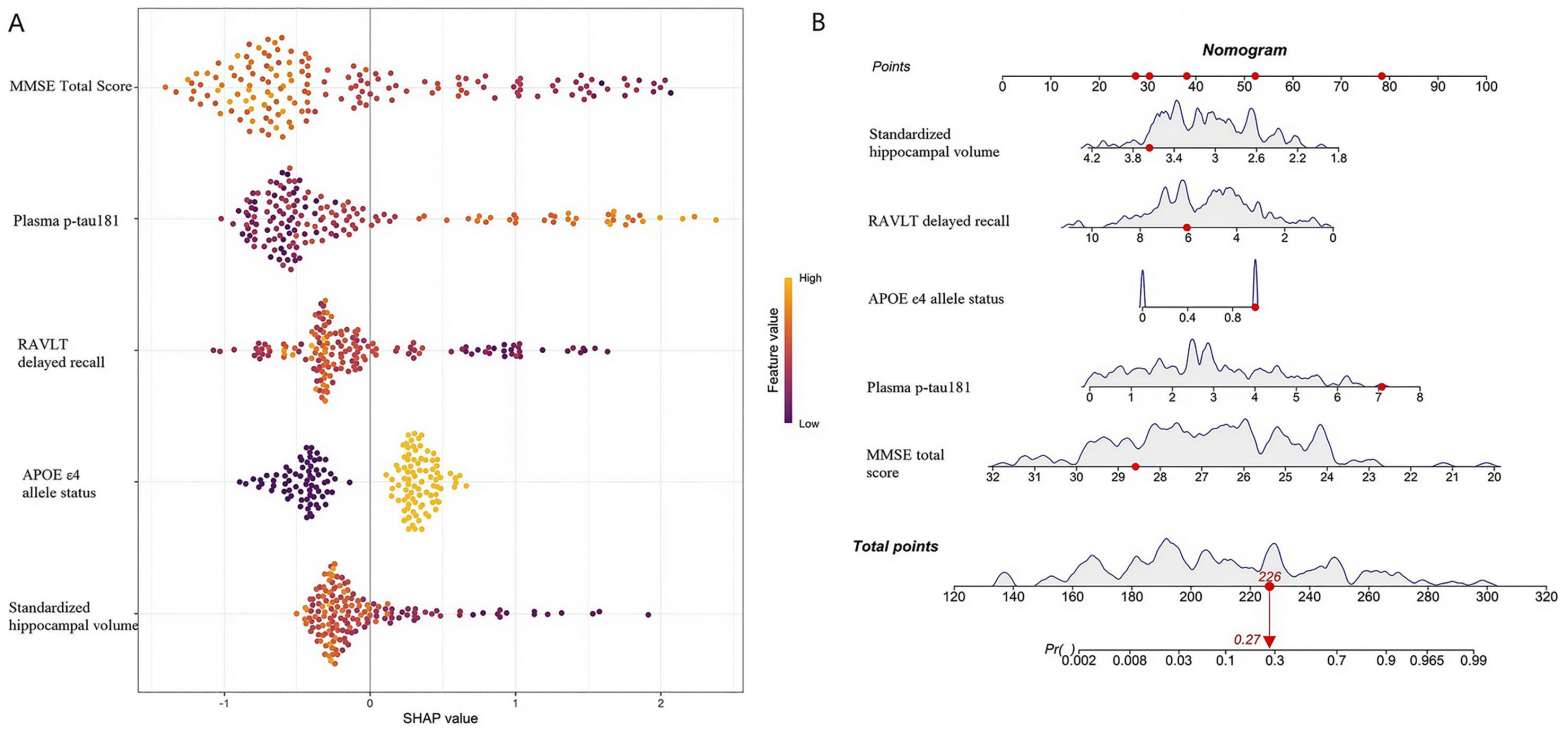
SHAP feature importance plot **(A)** and nomogram **(B)**.

SHAP values were employed to assess the interpretability of the highest AUC (logistic regression) from a global perspective, based on the five core variables selected by LASSO regression. The SHAP feature importance plot (Figure 5A) displayed the following ranking of variable contributions to the prediction outcome: normalized hippocampal volume > plasma p-tau181 > MMSE total score > RAVLT delayed recall > APOE ε4 allele status. Furthermore, a nomogram was constructed (Figure 5B) to visually depict the contribution of each variable to the risk of disease progression and the corresponding predicted probability based on the total points, thereby facilitating individualized clinical assessment. Both SHAP analysis and nomogram construction were performed using the training set data to ensure interpretability and model transparency.

## Discussion

The early prediction of AD is crucial for delaying or even preventing its onset and progression (8). In this study, approximately one-quarter (80/320, 25%) of the entire cohort comprising individuals with preclinical AD and AD-related MCI exhibited cognitive decline during the follow-up period (9, 10). Current clinical practice lacks reliable tools capable of integrating multidimensional information to quantitatively assess individual progression risk (11). Innovatively, this study integrated neuroimaging (hippocampal volume), an emerging plasma biomarker (p-tau181), cognitive assessments, and genetic risk. Feature dimensionality reduction was performed via LASSO regression, leading to the construction and comparison of multiple machine learning prediction models (12, 13). The results indicate that the model built upon five core variables exhibits favorable predictive performance. Specifically, the logistic regression model demonstrated robust discriminatory ability in both the training set and the independent validation set (AUCs of 0.849 and 0.818, respectively). All models showed good calibration and positive net clinical benefit, suggesting their potential for translation into clinically useful tools (14). SHAP analysis further enhanced model interpretability, identifying the neurodegeneration marker (hippocampal volume) and the tau pathology marker (plasma p-tau181) as the most important predictors. This provides a critical perspective for understanding the core pathological processes driving progression in preclinical AD.

The five core predictors identified in this study encompass multiple dimensions, including cognitive function, brain structure, tau pathology, and genetic susceptibility, thereby systematically characterizing the risk profile for AD progression (15, 16). First, the standardized hippocampal volume, as the strongest predictor, directly reflects key neurodegenerative changes in early AD, with its degree of atrophy closely correlating with the rate of cognitive decline. Second, plasma p-tau181, as a highly accessible blood biomarker for tau pathology, demonstrated predictive value independent of hippocampal volume. This indicates that the activity level of tau pathology is another core driver of disease progression (17). The APOE ε4 allele, as the most significant genetic risk factor, significantly increased progression risk in carriers, underscoring the fundamental role of genetic background in the disease’s natural history. Furthermore, the baseline MMSE total score and the RAVLT delayed recall score, representing cognitive reserve and memory function, exhibited a protective effect against clinical progression, with their preserved levels associated with delayed decline (18, 19). Notably, in the multifactor model, SUVR failed to show independent predictive value. This may suggest that within the preclinical/early AD population where Aβ pathology has already reached a positive threshold, further Aβ accumulation has a weaker predictive effect on short-term cognitive changes. Combined with the collinearity diagnosis and the sensitivity analysis of model performance comparison, Aβ-PET SUVR had a moderate correlation with hippocampal volume and plasma p-tau181, and the efficacy of the 6-variable model including it was not improved. Therefore, tau pathology and neurodegeneration become more direct short-term predictive indicators, which is highly consistent with the “cascade” pathological hypothesis of AD—that is, Aβ deposition is the initiating factor of AD pathology, while tau pathology and neurodegeneration are the core factors driving the progression of clinical symptoms. Although hippocampal volume is a classic indicator in AD research, this study is the first to quantitatively confirm its core predictive value and synergistic effect with plasma p-tau181 in the multimodal fusion model of Chinese Aβ-positive early AD populations, which is not a simple rediscovery but provides empirical evidence of the Chinese population for the clinical application of this classic indicator. This provides a critical perspective for understanding the core pathological processes driving progression in preclinical AD, and combined with the technological developments in the AD field from 2023 to 2026 (such as plasma p-tau subtype detection, multimodal image fusion) and the 2024 International Working Group AD clinical-biomarker diagnostic criteria, it further clarifies the core role of tau pathology and neurodegeneration in the early progression of AD, which is highly consistent with the recent research progress in the field (4, 20, 21).

Methodologically, this study adopted a rigorous modeling pipeline with traditional statistical methods as the foundation and machine learning methods as supplementary verification (22), without blindly using complex machine learning algorithms, which not only ensured the statistical transparency and scientific depth of the study, but also realized multi-method verification of the model’s predictive efficacy. LASSO regression effectively selected a parsimonious and robust set of core variables, thereby avoiding overfitting. By comparing machine learning models based on different principles, we found that while logistic regression performed robustly, ensemble learning models like random forest also demonstrated comparable performance. In this study, the logistic regression model outperformed other complex machine learning models, and the core reason is not that the complex models failed to achieve optimal tuning, but two points: first, the medium sample size of 320 cases in this study made complex models prone to overfitting, while the logistic regression model has low complexity and low overfitting risk, which is more suitable for the sample size of this study; second, nonlinear tests showed that the relationship between the five core predictors and early AD progression is mainly linear with no obvious nonlinear interaction, so the advantage of complex models in capturing nonlinear relationships cannot be exerted. This also suggests that simple linear models have higher practical value in clinical prediction studies with medium sample size and linear variable-outcome relationships (23). Decision curve analysis confirmed the clinical utility of the model across a wide range of threshold probabilities. This indicates that using this model to screen high-risk individuals for intervention yields a higher net benefit than strategies of either intervening in all or no individuals (24). Through SHAP analysis and nomogram visualization, complex model predictions were translated into intuitive individual risk scores, substantially enhancing the model’s clinical applicability and physician trust.

The innovation of this study lies in its systematic integration, for the first time in a domestic cohort, of multimodal biomarkers—including Aβ-PET, structural MRI, and plasma p-tau181—alongside genetic information, to construct and validate a prediction model for progression in preclinical AD. This provides a vital tool for early, precise risk assessment within the Chinese population.

Despite the above findings, this study still has several limitations. First, this study is a single-center, retrospective study with a relatively limited sample size, and has not been validated in independent external datasets such as ADNI. External validation through independent datasets is an essential prerequisite before the clinical application of this model, which can eliminate the selection bias caused by single-center design and confirm the predictive accuracy of the model in different clinical settings, geographic regions and biomarker detection platforms. The external generalizability of the model thus requires further validation in future multi-center, large-sample, prospective cohorts, and we will also focus on verifying the consistency of hippocampal volume as the most influential predictor in independent cohorts with different demographic and clinical characteristics. Second, the follow-up duration might not have captured the complete progression trajectory for all individuals. The model’s efficacy for predicting very long-term (e.g., 5–10 years) progression risk remains to be observed. Third, the model did not incorporate potentially influential dynamic variables, such as lifestyle factors or comorbidities. Future research will focus on conducting multi-dimensional external validation work with specific and feasible plans: we will cooperate with 3-4 Alzheimer’s disease research centers of tertiary grade A hospitals in China to carry out multi-center validation and collect homogeneous data of no less than 500 Aβ-positive early AD populations; conduct cross-platform validation for different detection platforms to evaluate the robustness of the model in plasma p-tau181 detection reagents from different manufacturers and hippocampal volume detection data of 3.0 T MRI from different models; and carry out 3–5 years of prospective cohort validation to verify the predictive value of the model for long-term disease progression. In addition, we will explore the inclusion of data from additional dimensions (e.g., connectomics, other novel plasma biomarkers) and attempt to utilize more complex deep learning models to uncover deeper predictive features, and integrate dynamic variables such as lifestyle and comorbidities to iteratively optimize the model, so as to further improve its predictive performance and clinical applicability. Of note, the primary outcome—cognitive progression—was defined as a transition from preclinical AD to MCI or dementia, or from MCI to dementia, rather than a uniform continuous decline across a single clinical stage. This composite definition, while clinically meaningful and aligned with real-world practice, introduces heterogeneity in the baseline severity and progression trajectories among participants, which may affect the generalizability of the model to populations defined by a single-stage transition. Thus, this operational definition of progression should be considered another limitation of the study.

In summary, this study constructed and validated a multimodal biomarker-based prediction model for progression in preclinical AD. This model integrates key information from cognitive, imaging, blood, and genetic domains, demonstrating good predictive performance, calibration, and clinical utility. The research not only confirms the central role of hippocampal atrophy and plasma p-tau181 in predicting short-term progression but also renders the prediction logic transparent through interpretability analysis. This tool holds promise for assisting clinicians and researchers in identifying high-risk individuals, providing a quantitative basis for early targeted interventions and clinical trial enrollment, thereby advancing AD prevention and treatment toward precision medicine.

## Data Availability

The original contributions presented in the study are included in the article/Supplementary material, further inquiries can be directed to the corresponding author.
