# Supplementary material for: Integrating neuroimaging and plasma biomarkers to predict preclinical Alzheimer’s disease progression
Source: Front Neurol. 2026 May 5;17:1801239. doi: 10.3389/fneur.2026.1801239 (PMC13185224; doi:10.3389/fneur.2026.1801239)
Supplement: Supplementary file 1 [file Table_1.DOCX]

**Supplemental Table 1**. Comparison of baseline characteristics between the disease progression and stable groups in the training set

| Variables | Disease Stable Group (n=168) | Disease Progression Group (n=56) | *t/χ²* | *P* |
| --- | --- | --- | --- | --- |
| Age (years) | 71.10±5.80 | 72.00±6.10 | 0.993 | 0.322 |
| Sex |  |  | 0.394 | 0.531 |
| Male | 71(42.26) | 21(37.50) |  |  |
| Female | 97(57.74) | 35(62.50) |  |  |
| Years of education | 13.00±3.20 | 12.60±3.40 | 0.798 | 0.426 |
| History of hypertension (%) |  |  | 0.215 | 0.643 |
| Yes | 87(51.79) | 31(55.36) |  |  |
| No | 81(48.21) | 25(44.64) |  |  |
| History of diabetes (%) |  |  | 0.083 | 0.773 |
| Yes | 33(19.64) | 12(21.43) |  |  |
| No | 135(80.36) | 44(78.57) |  |  |
| Family History of AD (%) |  |  | 0.178 | 0.674 |
| Yes | 49(29.17) | 18(32.14) |  |  |
| No | 119(70.83) | 38(67.86) |  |  |
| Clinical Stage (%) |  |  | 2.889 | 0.089 |
| Preclinical AD (Aβ+, Cognitively Normal) | 85(50.60) | 21(37.50) |  |  |
| MCI due to AD | 83(49.40) | 35(62.50) |  |  |
| MMSE Total Score | **27.30±1.80** | **25.40±2.10** | 6.554 | 0.001 |
| ADAS-Cog13 Total Score | 12.00±4.00 | 13.20±4.80 | 1.846 | 0.066 |
| RAVLT Delayed Recall | **5.60±1.90** | **4.00±2.00** | 5.386 | 0.001 |
| TMT-b Time, s | 123.80±43.50 | 130.90±50.10 | 1.017 | 0.310 |
| Aβ-PET SUVR | **1.20±0.17** | **1.31±0.19** | 4.070 | 0.001 |
| Standardized hippocampal volume , mL | **3.23±0.42** | **2.82±0.45** | 6.214 | 0.001 |
| Medial Temporal Lobe Cortical Thickness, mm | 2.67±0.26 | 2.62±0.30 | 1.198 | 0.232 |
| Mean Global Cortical Thickness, mm | 2.51±0.15 | 2.48±0.16 | 1.275 | 0.204 |
| White Matter Hyperintensity Volume, mL | 1.04±0.51 | 1.08±0.55 | 0.498 | 0.619 |
| Plasma p-tau181, pg/mL | **2.52±1.36** | **3.78±1.52** | 5.827 | 0.001 |
| Plasma Aβ42/40 Ratio | 0.05±0.01 | 0.05±0.01 | 0.001 | 1.000 |
| Plasma NfL, pg/mL | 18.50±7.60 | 19.60±8.70 | 0.904 | 0.367 |
| Plasma GFAP, pg/mL | 182.30±84.10 | 195.00±91.50 | 0.957 | 0.340 |
| APOE ε4 allele status |  |  | 15.583 | 0.001 |
| Carrier (%) | **78(46.43)** | **43(76.79)** |  |  |
| Non-carrier (%) | 90(53.57) | 13(23.21) |  |  |
| Polygenic risk score | 0.10±0.93 | 0.18±0.98 | 0.550 | 0.583 |

Abbreviations: AD, Alzheimer's disease; Aβ, amyloid-beta; MCI: mild cognitive impairment; MMSE, Mini-Mental State Examination; ADAS-Cog13, Alzheimer’s Disease Assessment Scale-Cognitive Subscale 13-item version; RAVLT, Rey Auditory Verbal Learning Test; TMT-b: Trail Making Test part B; Aβ-PET SUVR: amyloid-beta positron emission tomography standardized uptake value ratio; p-tau: phosphorylated-tau; NfL, neurofilament light chain; GFAP, glial fibrillary acidic protein; APOE , apolipoprotein E.
